# Voltage-Gated Calcium Channel Antibody-Induced Oropharyngeal Dysphagia Presenting as a Paraneoplastic Neurological Complication in Breast Cancer

**DOI:** 10.7759/cureus.13677

**Published:** 2021-03-03

**Authors:** Razwana Khanam, Ibrahim S Fanous, Eman N Fadhel, Tara Hyder, Adam Brufsky

**Affiliations:** 1 Internal Medicine, University of Pittsburgh Medical Center, McKeesport, USA; 2 Family Medicine, University of Pittsburgh Medical Center, McKeesport, USA; 3 University of Pittsburgh Physicians, University of Pittsburgh Medical Center, Pittsburgh, USA; 4 Hematology/Oncology, Magee Women's Hospital, University of Pittsburgh Medical Center, Pittsburgh, USA

**Keywords:** invasive ductal cell carcinoma, paraneoplastic syndromes, oropharyngeal dysphagia, voltage-gated calcium channels

## Abstract

Paraneoplastic neurologic syndromes (PNS) are a group of disorders characterized by an autoimmune response against the nervous system due to cross-reactivity between malignant and normal neural tissue. The most commonly associated malignancies include small cell lung cancer, ovarian cancer, breast cancer, and lymphoma. Multiple PNS have been reported including paraneoplastic cerebellar degeneration, retinopathy, sensorimotor peripheral neuropathy, encephalopathy, opsoclonus-myoclonus syndrome, and stiff-person syndrome.

We report a case of a 67-year-old woman with breast cancer who presented with a history of progressive oropharyngeal dysphagia as a paraneoplastic neurologic complication. She was diagnosed with invasive ductal carcinoma, nuclear grade 3 with moderate peritumoral lymphoid infiltrate. Hormone receptors were weakly positive for estrogen receptor (ER) (H score 15), weakly positive for progesterone receptor (PR) (H score 30), and negative for human epidermal growth factor receptor 2 (HER-2/NEU). The patient underwent a localized segmental mastectomy but declined any further adjuvant treatment. Three years after being diagnosed with invasive ductal carcinoma of the breast, she developed progressive oropharyngeal dysphagia that warranted percutaneous endoscopic gastrostomy (PEG) tube placement. Testing for onconeural antibodies was positive for voltage-gated calcium channel antibody. An extensive workup was negative for any alternative etiology that would explain her neurological symptoms. The patient declined further treatment and eventually succumbed to her illness.

## Introduction

Paraneoplastic neurologic syndromes (PNS) are uncommon in solid tumors. They are less frequently seen in association with breast cancer compared to other cancer types such as lung cancer or head and neck cancers. It is unclear whether certain patients’ subsets, receptor typing, or histological patterns of breast cancer predispose them to develop PNS [1].

In 2004, an international panel of neurologists established a set of guidelines that would assist in the diagnosis and classification of PNS into two categories: “definite” or “possible” [2]. The diagnostic criteria for “definite” include a “classical” neurological syndrome with onconeural antibodies or cancer that occurs within five years of the diagnosis of neurological symptoms. The term “classical syndrome” applies to those neurological syndromes that have a strong association with cancer. Some of these syndromes include encephalomyelitis, limbic encephalitis, subacute cerebellar degeneration, opsoclonus-myoclonus, Lambert-Eaton myasthenic syndrome, and dermatomyositis. Furthermore, a diagnosis of PNS requires ruling out other potential causes for the neurological symptoms.

Paraneoplastic cerebellar degeneration (PCD) is one of the most common subtypes of PNS affecting the central nervous system. It has been reported in approximately one percent of cancers and has been most commonly associated with small cell lung cancer (SCLC) [3]. Patients with PCD can have a wide range of clinical presentations including truncal ataxia, nystagmus, vertigo, dysarthria, dysphagia, and diplopia. Cognitive disorders and psychiatric symptoms have also been observed [4]. The clinical manifestations are usually bilateral with an asymmetrical distribution [5]. The patient can also present with stroke-like symptoms, tremors, and disorders of speech [6,7]. An integral part of diagnosing PCD requires the exclusion of other possible etiologies such as brain metastases, infections, metabolic derangements, vascular, or drug toxicities.

Historically, PCD has been associated with multiple onconeural antibodies including anti-Yo, anti-Hu, anti-Tr, anti-Ri, anti-mGluR1, and anti-voltage-gated calcium channel (anti-VGCC) antibodies. The anti-Yo antibody has been the most commonly reported antibody in association with PCD [5]. VGCC antibodies have been reported in association with Lambert-Eaton syndrome. VGCC antibodies have been reported less frequently in paraneoplastic syndromes. Most of these cases have been in association with SCLC [8]. Approximately 40% of patients with subacute onset cerebellar degeneration, usually with SCLC, are also positive for the VGCC antibody [3,9-13].

Testing for onconeural antibodies can be done on serum or cerebrospinal fluid (CSF). In one study, 62 cases of PCD were identified and fully characterized between 1966 and 1990. The anti-Yo antibody was detected in the analysis of peripheral blood and CSF samples. This meant that patients did not have to undergo invasive CSF testing in order to make the diagnosis of PCD [14]. Here, we present a case of VGCC antibody-mediated oropharyngeal dysphagia presenting as a paraneoplastic neurological complication in association with invasive ductal carcinoma of the breast.

## Case presentation

**Figure 1:**
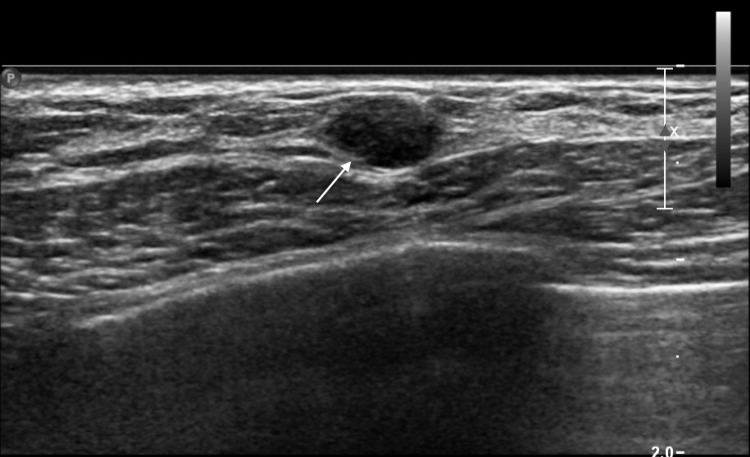
Oval hypoechoic circumscribed mass measuring 0.6 x 0.4 x 0.6 cm is identified in the 01:00 region (arrow) 7 cm from the nipple

The patient is a 67-year-old woman who was found to have a mass in her left breast on routine mammography. Ultrasonography revealed a 0.6 × 0.4 × 0.6 cm mass (Figure 1). A biopsy revealed invasive ductal carcinoma, nuclear grade 3 with moderate peritumoral lymphoid infiltrate. The tumor was weakly positive for estrogen receptor (ER) (H score 15), weakly positive for progesterone receptor (PR) (H score 30), and human epidermal growth factor receptor 2 (HER2)-negative. The tumor proliferation index Ki-67 was 70%.

The patient had a past medical history significant for HIV that was well controlled on zidovudine, lamivudine, and efavirenz. She also had ischemic cardiomyopathy with a reduced ejection fraction of 20%, dyslipidemia, and essential hypertension. Although she had a positive history of breast cancer, she declined BRCA screening. She subsequently underwent left-sided localized segmental mastectomy with sentinel node biopsy. Surgical margins were negative, and four sentinel lymph nodes were negative for tumor as well. She was pathologically staged as pT1bN0. The patient declined any further adjuvant therapy. She continued with an annual surveillance mammogram to detect any evidence of recurrence.

Three years later, the patient started complaining of dysphagia to both liquids and solids with no reported odynophagia. A modified barium swallow demonstrated findings consistent with severe oropharyngeal dysphagia. The patient’s symptoms led to malnutrition and failure to thrive and a decision was made to place a percutaneous endoscopic gastrostomy (PEG) tube for nutrition.

A workup was done to identify the cause of her oropharyngeal dysphagia. A flexible laryngoscopy demonstrated normal vocal cords with no anatomical abnormalities to explain her symptoms. The acetylcholine receptor antibodies (AchR-Ab), including binding, blocking, and modulating antibodies, were negative, which ruled out myasthenia gravis. Brain MRI was able to rule out multiple sclerosis, progressive multifocal leukoencephalopathy, and brain metastasis. CSF analysis showed oligoclonal bands that were deemed non-specific at that time. Investigations for potential infectious causes were negative for syphilis and Lyme disease. The patient was eventually discharged without determination of the underlying etiology for the oropharyngeal dysphagia. 

The patient was readmitted a month later to the intensive care unit for management of acute respiratory failure secondary to pneumonia and sepsis. A CT chest, abdomen and pelvis was negative for any metastatic lesion. During that admission, testing for serum onconeural antibodies was positive for VGCC antibodies (>30 pmol/L).

Based on the guidelines established for the diagnosis of PNS, the patient's clinical presentation appeared consistent with definite PNS. The classification of PNS as definite requires a non-classical syndrome with onconeural antibodies (well-characterized or not) and cancer that develops within five years of the diagnosis of the neurological disorder. The patient's symptoms of dysphagia were felt to be a non-classical manifestation of PNS, which was further supported by the identification of the onconeural antibody for VGCC and a negative workup for other possible etiologies.

The patient declined any further treatment. She was made comfortable as per her wishes and the family members. The patient succumbed to her disease shortly.

## Discussion

We are presenting a case of a patient with oropharyngeal dysphagia as a paraneoplastic neurologic complication of breast cancer. Our case report confirms the importance of considering PNS when investigating breast cancer patients presenting with neurological symptoms of unknown etiology. In most cases of PNS, neurological symptom onset precedes a tumor diagnosis [15]. However, there have been reported cases of patients developing PNS despite treatment of primary breast cancer [16]. Other case reports showed improvement of the neurological symptoms after therapy of the primary tumor [17].

The presence of VGCC antibodies has been frequently found in association with Lambert-Eaton syndrome and SCLC. However, there are reported cases of VGCC antibodies with breast cancer that lead to various phenotypes ranging from muscle weakness, chorea, dysphagia, dysarthria, and even respiratory failure [18,19].

Given the rare incidence of PNS, treatment strategies have been based on limited case series and cohort studies. Treatment of PNS can be differentiated into the treatment of the underlying tumor and immunotherapy. Prompt initiation of tumor therapy should be instituted according to current clinical practice guidelines. Treatment of the underlying tumor enables stabilization or improvement of the neurological disorders [20].

There have been no reported cases of PNS in breast cancer patients improving with immunotherapy. However, immunosuppressive therapy has been used with varying degrees of success. Intravenous immunoglobulin (IVIG), steroids, and plasma exchange were more successful in the management of PNS in breast cancer patients. Other immunosuppressive agents used include cyclophosphamide, mycophenolate, and rituximab [1].

## Conclusions

PNS is a rare neurological complication in breast cancer, which makes developing guidelines for the diagnosis and treatment of these disorders challenging. More prospective studies and clinical trials are required to draw more evidence-based conclusions. However, PNS should be considered in the differential of patients presenting with unexplained neurological symptoms. Since diagnosing PNS can take time, we recommend the prompt initiation of treatment in cases with a high index of suspicion since it may help stabilize or improve the neurological disorder.
